# Bogarting that joint might decrease oral hpv among cannabis users

**DOI:** 10.3747/co.v16i6.429

**Published:** 2009-12

**Authors:** S.R. Zwenger

**Keywords:** Oral hpv, cannabis, cancer

## Abstract

Human papilloma virus (hpv) has many known strains, two of the most well studied perhaps being the high-risk types 16 and 18. These strains have attracted more interest because they are known to disrupt tumour-suppressor genes that control the cell cycle, rendering those genes less effective at keeping cell division in check.

Within the last decade, an increase in oral hpv–linked cancers of the throat and tongue has been attributed to exposure and contraction of hpv through oral sex, most notably in younger people. An understudied and arguably equal contributor to oral hpv infection might be indirect contact with an infected person. Presented here is a brief but important perspective on the relationship between cannabis use and oral cancer. The development of oral cancer is not a result of smoking cannabis *per se;* rather, it is hypothesized to be a result of contracting hpv through various forms of sharing and passing joints and other smoking apparatuses. Therefore, it is hypothesized that bogarting (and not passing) joints might decrease oral hpv among cannabis smokers. Future research should therefore investigate the prevalence of oral hpv in cannabis smokers to better understand its epidemiology.

## 1. INTRODUCTION

The American Cancer Society estimates that, in the United States, more than 6 million people contract human papilloma virus (hpv) each year and that nearly half are between 15 and 25 years of age. Oral sex with an hpv-infected person has been shown possibly to increase the likelihood of contracting oral hpv^1^. Expression of either E6 or E7 high-risk hpv (types 16 and 18) oncoprotein is known to interfere with the cell-cycle tumour suppressors p53 and pBR. Sustained expression of these oncoproteins is ultimately able to drive chromosomal mutations, which lead to cancerous cells^2^.

Patients treated for head-and-neck cancers can have a drastically degraded quality of life. For example, in many cases, treatment and surgeries can lead to increased depression, increased anxiety, and decreased marital quality, and can cause difficulty with daily tasks such as eating and speaking^3^. Chandu *et al.*^4^ assert that understanding the impact of oral cancer on patients can be better determined with the use of surveys that assess their health-related quality of life, and can also lead to better patient care. Previously, such surveys were used primarily in research settings.

The rapid rise in oral cancers among young people is expected to continue. This rise has largely been attributed to contraction of oral hpv through sexual behaviours (for example, oral sex). Additionally, Rose Ragin *et al.*^5^ showed that women with hpv-related cervical cancer had a higher chance of head-and-neck cancer. Using data from the National Cancer Institute’s Surveillance, Epidemiology, and End Results database, they found that women with cervical cancer had a greater chance of developing head-and-neck squamous cell carcinomas. The authors suggested that the head-and-neck cancers are caused by secondary infections from hpv. D’Souza *et al.*^6^ determined that oropharyngeal cancer and infection with oral hpv are strongly linked. They also suggest that oral hpv is often contracted through oral sex. However, Sok and Grandis^7^ stated that some of the routes of oral hpv infection are unknown. Regardless, many people have suggested that a vaccine against oral hpv might be important in preventing oral hpv infections^8^–^10^.

## 2. DISCUSSION

### 2.1 Oral HPV and Cannabis

Given these considerations, there has been much debate on whether cannabis has been a causative agent of oral cancers. As with hpv-linked cancers, studies have tried to find a relationship between cannabis smoking and a variety of types of cancers. For example, Almadori *et al.*^11^ postulated a link between cannabis smoking and tongue carcinomas. Hashibe *et al.*^12^ conducted a study on 2252 subjects, including people with and without cancer, and found no relationship between cannabis use and lung and aerodigestive tract cancers. Some authors have hypothesized that cannabis use might be a cause of transitional cell carcinoma, a type of bladder cancer typically associated with tobacco smokers^13^,^14^. Hall *et al.*^15^ reviewed the relationships between cancer and cannabis, including its possible role as a cause of cancer and its use to relieve pain in cancer patients. Others have reviewed cannabis use and the probability of developing cancer^16^. In a more recent study, Aldington *et al.*^17^ investigated the relationship between cannabis and head-and-neck cancer and found little or no correlation.

However, in all of the above-mentioned studies and reviews, hpv as a possible factor in the development of cancer was not discussed. The importance of understanding the role that cannabis smoking might play in hpv-related cancers is difficult to overstate. Indeed, a recent report by the World Health Organization notes that countries must begin to take action toward dealing with oral cancer prevention^18^. If cannabis use is indeed a major contributor to spreading oral hpv, steps should be taken by governments to educate their citizens to help mitigate oral cancer rates.

Worldwide consumption of cannabis has been documented by the United Nations *World Drug Report*^19^. Based on the report’s estimates, 165,600,000 people used cannabis in 2006. North America and Europe accounted for about 70 million of those users, both regions being above the global average. A report by Leatherdale *et al.*^20^ states that nearly half of Canadian adults 18 years of age or older have tried cannabis in spite of its illegality. However, some countries have recognized the medicinal properties of this plant product and approved it for medical use, which may also contribute to the observed increase in use. For example, part of the increased cannabis use in Canada is based on the fact that, although cannabis is classified as an illegal drug, individuals who are living with a qualifying debilitating illness can legally obtain it through Health Canada’s Medical Marihuana Access Division. The possibility that hpv can be passed among cannabis users is therefore significant.

The human papilloma virus can be found on the surface of the lips, the lining of the mouth, on the tongue, and so on. Therefore, as an infected person places a cannabis cigarette or smoking device on their lips, they simultaneously deposit viral particles on the device. The hpv remains there until another user places the same area of the smoking device onto their lips. This scenario could easily have continued for many years as a possible method of oral hpv transmission.

Although partaking in cannabis smoking may be an individual process, cannabis smokers are also known to pass and share their cannabis freely. For instance, various studies have found that, although some cannabis users smoke alone, others share with close friends or at parties, making smoking a social activity^21^,^22^. Dunlap *et al.*^23^ observed that sharing cannabis wrapped in a cigar shell (called a “blunt”) often occurs among large groups of people.

### 2.2 Needed Research

Gillison and Lowy^24^ discussed some ways in which people might contract hpv and postulated reasons that vaccines might be useful in controlling hpv-related cancers in later life. They cited use of alcohol and tobacco (because of the carcinogenic nature of those substances) as additional contributors to the likelihood that these types of cancers may develop. The mention of tobacco is interesting, because it, too, may serve as an indirect route of transmission. For example, Knishkowy and Amitai^25^ discuss tobacco smoking through water pipes, a practice that is very common in most Middle Eastern countries. They warn of the rise of “hooka bars” and their negative impact on the health of young adults, because this group seems to be the main one using tobacco water pipes. Although they mention that sharing of these pipes can lead to a variety of diseases, hpv is not mentioned. Still, sharing tobacco-smoking devices could also serve as an additional route of oral hpv contraction. To compound matters further, it not exactly known how easily oral hpv can be transmitted between individuals (for example, through sharing water bottles in sports or through casual kissing).

With such uncertainty, one would hope that a rapid assay for oral hpv would soon be available. Such a test might prove useful in a clinical setting, for research purposes, or for personal knowledge. Currently, in individuals positive for oral hpv, hpv antibodies can be detected in the mucosal areas. Marais *et al.*^26^ described a study in which they used an enzyme-linked immunosorbent assay to detect hpv antibodies from oral swabs. This same method might be desirable in a controlled study of cannabis users in future research. A study of this nature could provide a relatively rapid analysis of cannabis users and rates of hpv (for example, testing positive or negative as oral hpv carriers). Additionally, because cannabis use is often higher among men, higher rates of oral cancer might be observed in men than in women. Studying the frequency of cannabis use might also result in important findings such as an increased likelihood of users testing positive for oral hpv. Comparing individuals that share cannabis in group settings with those who consume cannabis alone might provide insight into more definitive patterns of oral hpv and thus help to predict the likelihood of head-and-neck cancers developing in later life.

## 3. CONCLUSIONS

It certainly may not be the cannabis smoke that causes oral cancers in heavy cannabis users. Most people who have ever smoked cannabis have most likely done so by sharing a rolled cigarette or pipe in a group setting. The sharing and passing of these smoking devices from an oral hpv-infected individual to an uninfected individual could easily provide a route of transmission for the virus between users. Frequency and setting should therefore be considered two major factors that might contribute to the likelihood of acquiring oral hpv. Thus, the relationship between cannabis and various cancer types might not be from cannabis use itself, but rather from contracting high-risk types of oral hpv that lead to cancer in later life.
